# Supplementary material for: Surface Functionalization with Polyethylene Glycol and Polyethyleneimine Improves the Performance of Graphene-Based Materials for Safe and Efficient Intracellular Delivery by Laser-Induced Photoporation
Source: Int J Mol Sci. 2020 Feb 24;21(4):1540. doi: 10.3390/ijms21041540 (PMC7073198; doi:10.3390/ijms21041540)
Supplement: Supplementary file 1 [file ijms-21-01540-s001.pdf]

Supplementary material

Surface functionalization with polyethylene glycol and polyethyleneimine improves the performance of graphene-based materials for safe and efficient intracellular delivery by laser-induced photoporation

Jing Liu[1], Chengnan Li[2], Toon Brans[1], Aranit Harizaj[1], Shana Van de Steene[3], Thomas De Beer[3], Stefaan De Smedt[1], Sabine Szunerits[2], Rabah Boukherroub[2], Ranhua Xiong[1], Kevin Braeckmans[1,4,#]

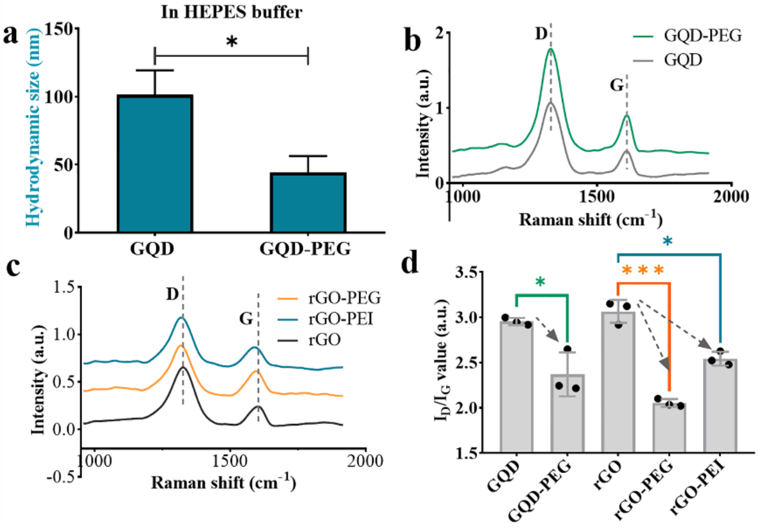

Figure S1. Raman spectra of GQD and GQD-PEG. **a.** Hydrodynamic size of GQD, and GQD-PEG as measured by DLS in HEPES buffer. **b.** Raman spectra of GQD and GQD-PEG and **c.** of rGO, rGO-PEG, and rGO-PEI. The dashed lines mark the position of the D (1326 cm^{-1}) and G bands (1610 cm^{-1}). **d.** The quantification of the I_D/I_G ratio. A statistical difference is found between GQD and GQD-PEG, rGO and rGO-PEG, and rGO and rGO-PEI, confirming successful modification of GQD with PEG and rGO with PEG and PEI. *P < 0.01, ***P < 0.001.

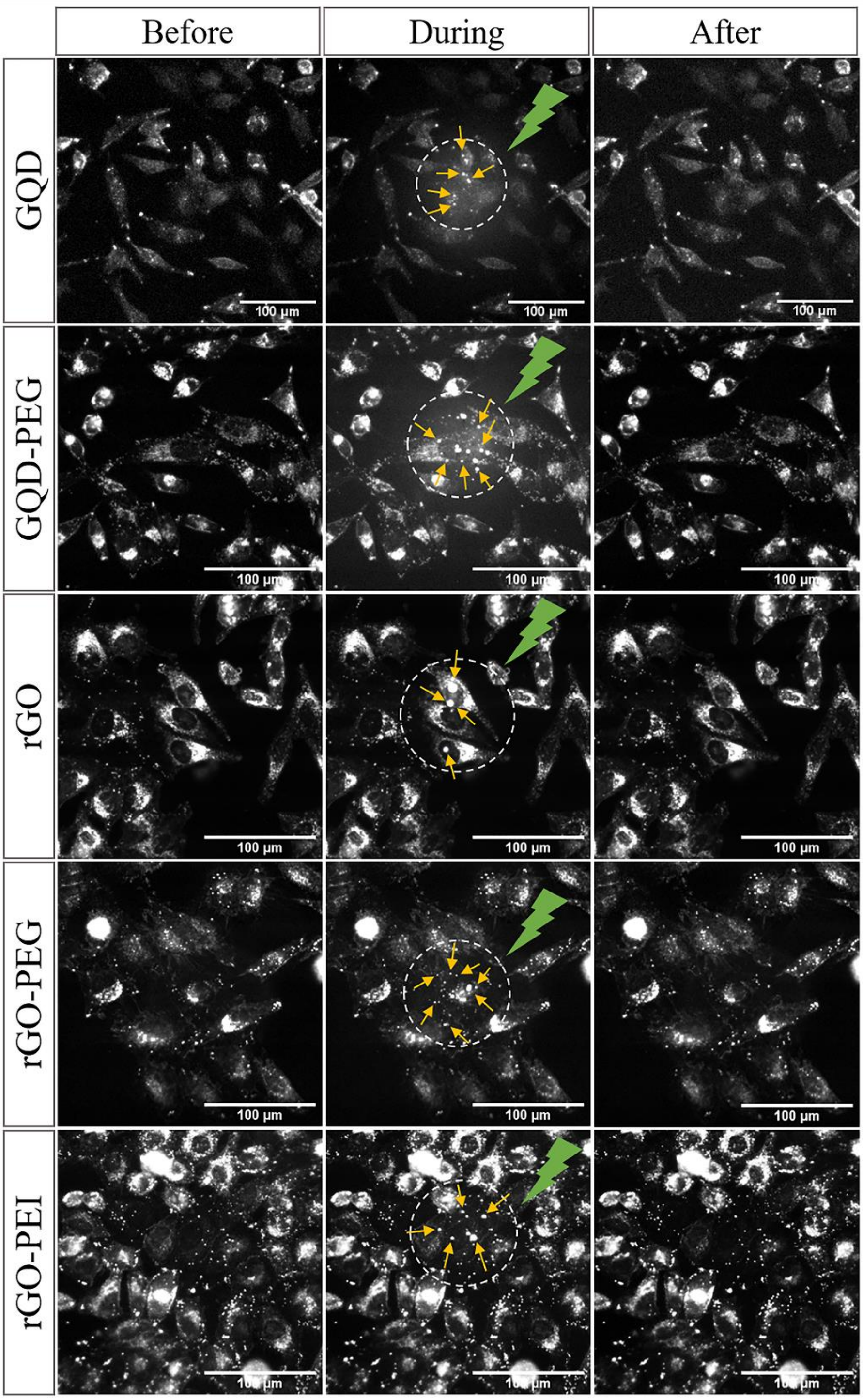

Figure S2. Dark field microscopy images of VNB formed at the HeLa cell membrane. (first column) HeLa cells are first incubated with graphene-based nanoparticles: GQD-PEG (first row), rGO (second row), rGO-PEG (third row) and rGO-PEI (fourth row). Note that individual particles are not visible in these images. (Middle column) A single 561-nm laser pulse of 7 ns is applied in the indicated circular region, triggering the formation of VNB which are visible as transient bright spots of light (some examples are indicated with yellow arrows). The scale bare is100 µm.

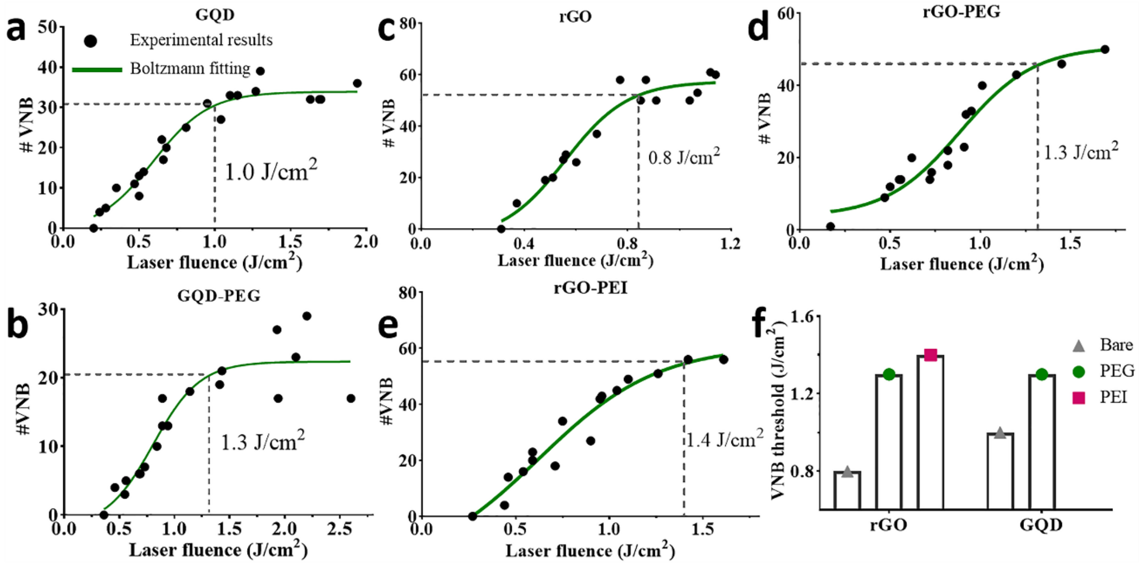

Figure S3. VNB formation threshold of GQD and rGO before and after functionalization with PEG and PEI. a. GQD, b. GQD-PEG, c. rGO, d. rGO-PEG and e. rGO-PEI. The black dots indicate the number of VNB that were observed for a given laser fluence. The green curves are a Boltzmann fit to the experimental data. The VNB formation threshold is marked in each graph with the dashed lines. f. Overview of the VNB threshold of rGO, rGO-PEG and rGO-PEI, showing that the VNB threshold has increased after functionalization.

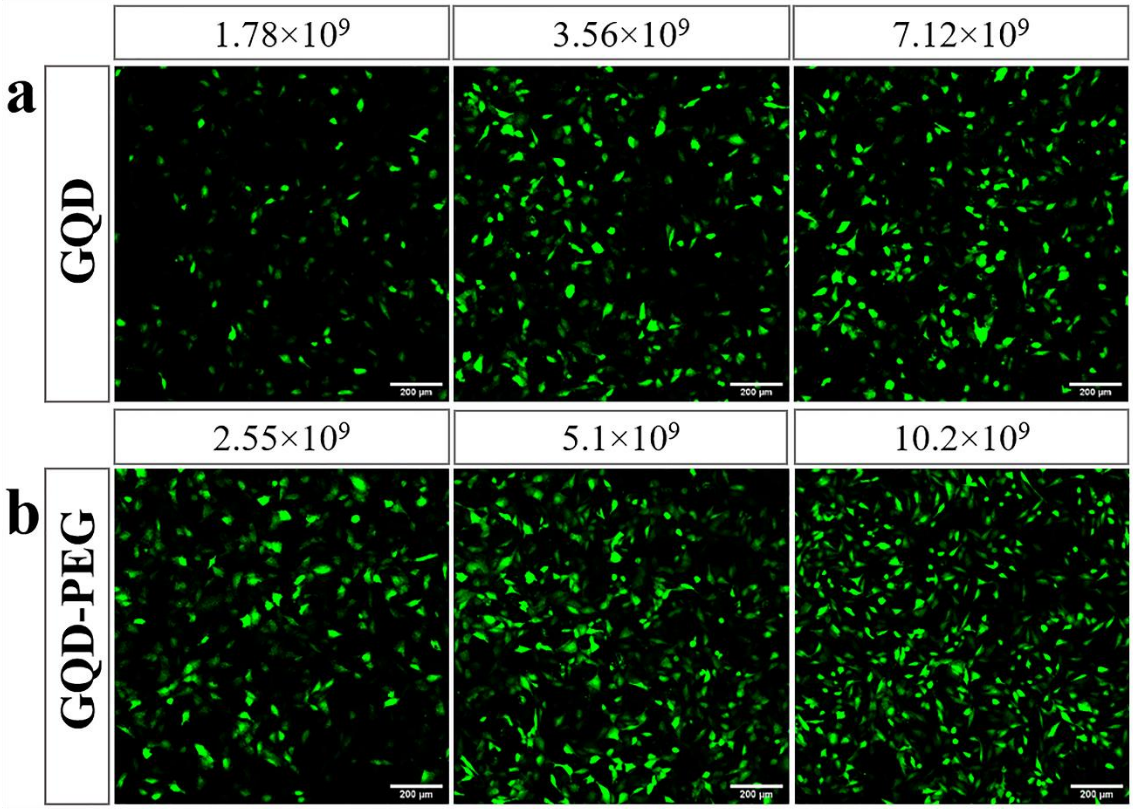

Figure S4. Confocal microscopy images of living HeLa cells after photoporation with FD10. HeLa cells are first incubated with a. GQD and b. GQD-PEG at the indicated concentrations (expressed as nps/mL). Cells are photoporated by pulsed laser treatment in the presence of 1 mg/ml FD10. After thorough washing of FD10, confocal images were recorded. The scale bares are 200 μm in all the images.

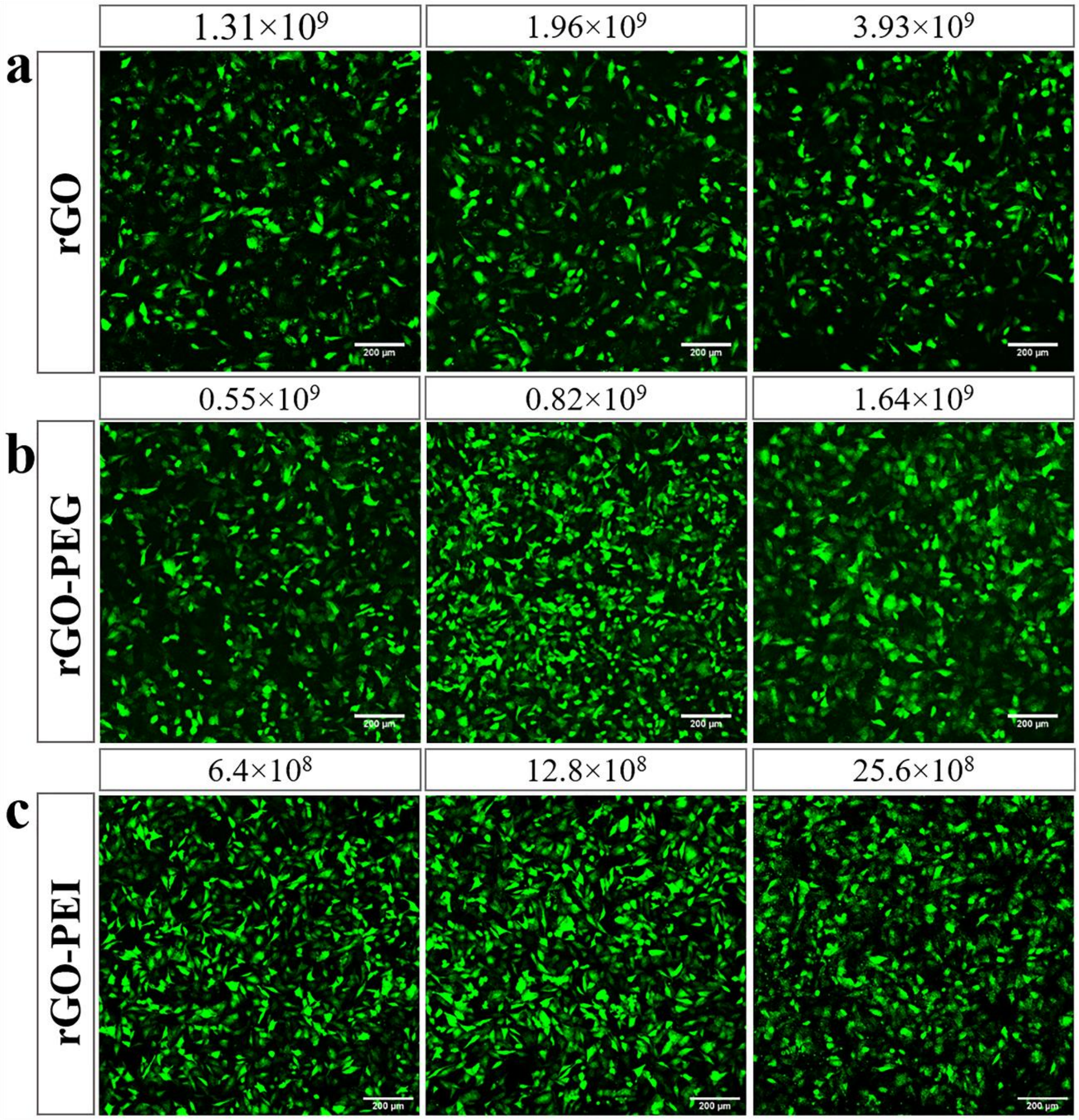

Figure S5. Confocal microscopy images of living HeLa cells labeled with FD10 by photoporation. HeLa cells are first incubated with a. rGO, b. rGO-PEG, and c. rGO-PEI at the concentrations indicated above each image (nps/mL). Cells are photoporated by pulsed laser treatment in the presence of 1 mg/ml FD10. After thorough washing of FD10, confocal images were recorded. The scale bares are 200 μm in all the images.

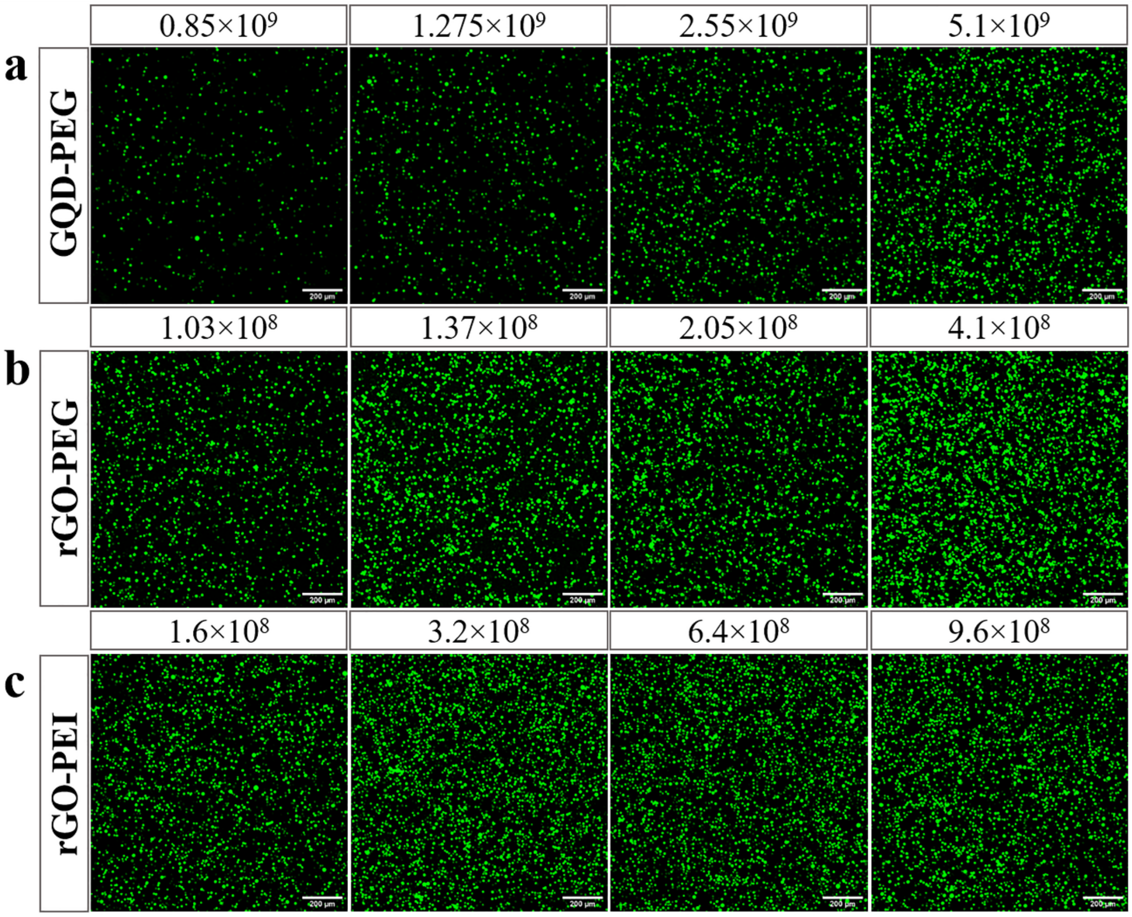

Figure S6. Confocal microscopy images of Jurkat cells labeled with FD10 by photoporation. Jurkat cells are incubated with a. GQD-PEG, b. rGO-PEG, and c. rGO-PEI at the indicated concentrations (nps/mL). Cells are photoporated by pulsed laser treatment in the presence of 1 mg/ml FD10. After centrifugation and washing to remove FD10, cells are re-suspended in fresh cell medium. Confocal microscopy images are recorded after cell sedimentation. The scale bares are 200 µm in all the images.

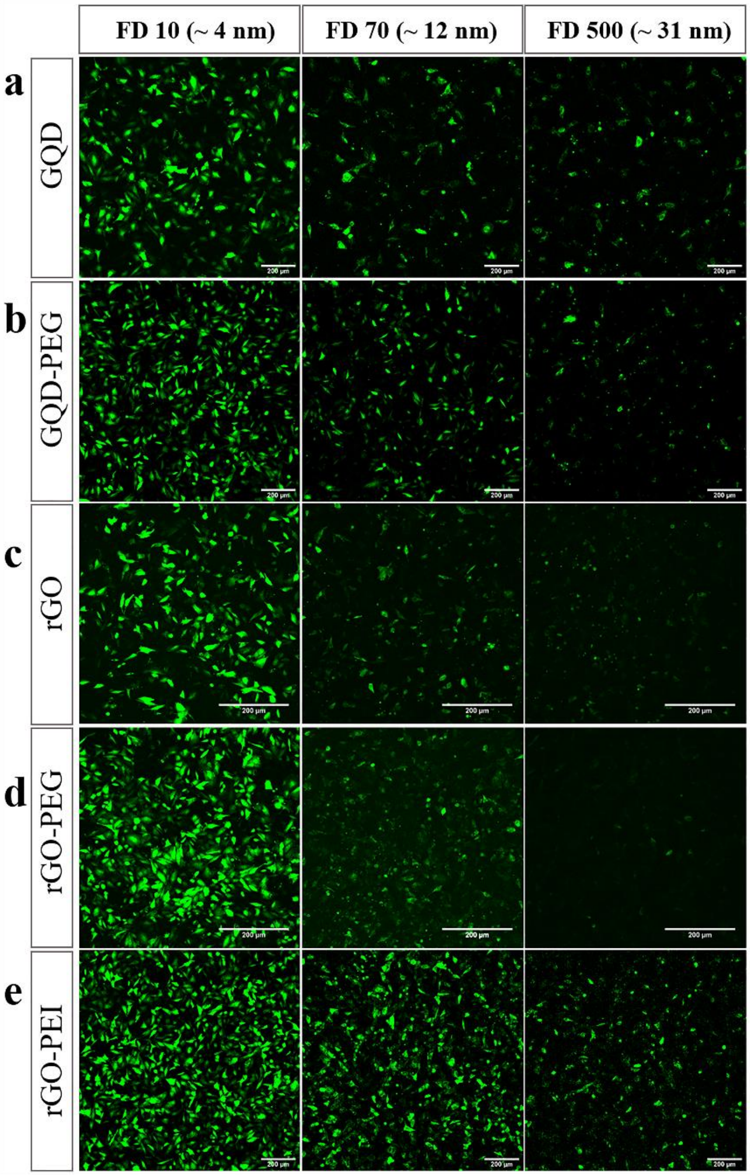

Figure S7. Confocal microscopy images of HeLa cells photoporated with FD10, FD70, and FD500. a-e. Laser treatment with 561 nm ns pulsed laser light. a. GQD, b. GQD-PEG, c. rGO, d. rGO-PEG, and e. rGO-PEI. Laser treatment with 800 nm ps pulsed laser irradiation using rGO-PEI for VNB generation. The scale bar is 200 μm.

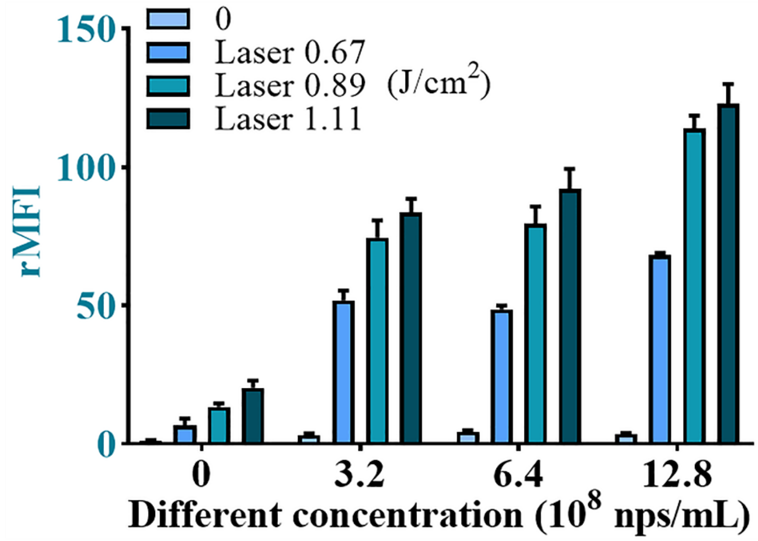

Figure S8. rMFI of FD10 photoporated with 800 nm laser light in HeLa cells as measured by flow cytometry. FD10 is delivered into cells by photoporation with different concentrations of rGO-PEI. 800 nm pulsed laser light (2 ps) is used for laser treatment at the indicated laser fluences.
